# Myotonia in a patient with a mutation in an S4 arginine residue associated with hypokalaemic periodic paralysis and a concomitant synonymous *CLCN1* mutation

**DOI:** 10.1038/s41598-019-54041-0

**Published:** 2019-11-26

**Authors:** Michael G. Thor, Vinojini Vivekanandam, Marisol Sampedro-Castañeda, S. Veronica Tan, Karen Suetterlin, Richa Sud, Siobhan Durran, Stephanie Schorge, Dimitri M. Kullmann, Michael G. Hanna, Emma Matthews, Roope Männikkö

**Affiliations:** 1 MRC Centre for Neuromuscular Diseases, Department of Neuromuscular diseases, UCL Queen Square Institute of Neurology, London, WC1N 3BG UK; 2 Department of Clinical and Experimental Epilepsy, UCL Queen Square Institute of Neurology, London, WC1N 3BG UK; 3 Department of Neurology and Neurophysiology, Guy’s and St Thomas’ NHS Foundation Trust, London, UK; 4 Present Address: Kinases and brain development laboratory, Francis Crick Institute, London, UK; 5 Present Address: UCL School of Pharmacy, UCL, London, UK

**Keywords:** Neuromuscular disease, Sodium channels, Chloride channels

## Abstract

The sarcolemmal voltage gated sodium channel Na_V_1.4 conducts the key depolarizing current that drives the upstroke of the skeletal muscle action potential. It contains four voltage-sensing domains (VSDs) that regulate the opening of the pore domain and ensuing permeation of sodium ions. Mutations that lead to increased Na_V_1.4 currents are found in patients with myotonia or hyperkalaemic periodic paralysis (HyperPP). Myotonia is also caused by mutations in the *CLCN1*gene that result in loss-of-function of the skeletal muscle chloride channel ClC-1. Mutations affecting arginine residues in the fourth transmembrane helix (S4) of the Na_V_1.4 VSDs can result in a leak current through the VSD and hypokalemic periodic paralysis (HypoPP), but these have hitherto not been associated with myotonia. We report a patient with an Nav1.4 S4 arginine mutation, R222Q, presenting with severe myotonia without fulminant paralytic episodes. Other mutations affecting the same residue, R222W and R222G, have been found in patients with HypoPP. We show that R222Q channels have enhanced activation, consistent with myotonia, but also conduct a leak current. The patient carries a concomitant synonymous *CLCN1* variant that likely worsens the myotonia and potentially contributes to the amelioration of muscle paralysis. Our data show phenotypic variability for different mutations affecting the same S4 arginine that have implications for clinical therapy.

## Introduction

The skeletal muscle sodium channel Na_V_1.4 conducts the key depolarizing current of action potentials, driving muscle excitability and contraction. Mutations in *SCN4A* that enhance the function of Na_V_1.4 are found in patients with myotonia (delayed muscle relaxation after contraction) due to spontaneous repetitive action potential firing – manifesting clinically as muscle stiffness or cramp – and/or periodic paralysis (PP) that presents as episodic muscle weakness or paralysis^1–4^. PP is usually associated with increased (Hyperkalaemic periodic paralysis, HyperPP) or decreased (Hypokalaemic periodic paralysis, HypoPP) serum potassium levels. All of these *SCN4A* disorders have autosomal dominant inheritance or can occur *de novo*.

Skeletal muscle excitability can also be compromised by mutations in other sarcolemmal ion channel genes^1,4^. Myotonia can be caused by mutations in *CLCN1* that reduce the function of the skeletal muscle chloride channel ClC-1 (myotonia congenita, MC). The most common cause of HypoPP are mutations in *CACNA1S* that encodes the skeletal muscle calcium channel Ca_V_1.1. In addition, mutations in *KCNJ2* that result in reduced function of the inwardly rectifying potassium channel Kir2.1 are associated with PP in Andersen-Tawil syndrome.

Na_V_1.4 channels are transmembrane proteins with modular organisation^5,6^. A pore domain allows the selective permeation of sodium ions and contains the activation gate of the channel and the docking site for an inactivation particle. Four voltage sensing domains (VSDs I-IV) respond to changes in membrane voltage and thereby control the opening and closing of the pore domain. The fourth transmembrane helix (S4) of each VSD contains several voltage-sensing arginine residues^5–7^.

Mutations that result in enhanced activity of the Na_V_1.4 pore domain are associated with sodium channel myotonia (SCM), paramyotonia congenita (PMC) and HyperPP^1–4^. Excess sodium inflow during myotonic repetitive action potential firing can eventually contribute to prolonged depolarization of the muscle, inactivation of Na_V_1.4 channels and subsequent inability of the muscle to contract. This presents as HyperPP and explains why some patients display overlapping clinical symptoms of myotonia and HyperPP. HypoPP is instead caused by mutations affecting voltage-sensing S4 arginines in either Nav1.4 or the related Ca_V_1.1 channel^3,4,8,9^. Mutation of these residues results in an aberrant leak current through the VSD^10^, a so-called gating pore current, which results in depolarization of the muscle particularly in hypokalaemic conditions, inactivation of Na_V_1.4 channels and PP. Consistent with distinct pathogenic mechanisms, myotonia is not a recognized feature of HypoPP.

We report a patient carrying a mutation affecting an S4 arginine, R222Q in Na_V_1.4, who presents predominantly with myotonia. Mutations affecting the same residue have previously been reported in patients with HypoPP^8,9^. Investigations revealed enhanced R222Q channel activation, and gating pore currents – although their amplitude in *Xenopus laevis* oocytes was smaller than for the HypoPP-associated mutations affecting the same residue. A concomitant synonymous *CLCN1* variant that is associated with recessive MC was also found in the patient. Our data detail the phenotypic and mechanistic variability of mutations affecting the same S4 arginine residue.

## Results

### Patient data

The proband is of Kurdish heritage with no known significant childhood or developmental abnormalities. At age 17 he complained of muscle stiffness and cramps affecting predominantly the legs, but also hands and jaw muscles. On clinical examination, eyelid, hand grip and gait myotonia were evident. Calf hypertrophy, and a history of severe bruxism with masseter hypertrophy were noted. Bilateral hip flexion strength was documented as Medical Research Council (MRC) strength score 4.

A presumed clinical diagnosis of MC was made and he was commenced on mexiletine with the dose uptitrated to 50 mg mane, 100 mg at mid-day, and 200 mg nocte as the patient preferred a variable dose. Higher doses were not tolerated at this stage due to dyspepsia. He considered this to be moderately effective, but continued to complain of stiffness and cramps with prominent falls. However, he noted that symptoms were considerably worse if he missed a dose of mexiletine. Follow-up appointments during his early 20 s consistently revealed muscle stiffness with falls but he also complained of weakness in his arms and legs, which he found difficult to quantify. Examination continued to demonstrate myotonia but no significant change in muscle strength.

At age 25 he described clearer episodic lower limb weakness that occurred after prolonged sitting, lasting approximately 5–10 minutes. He also noticed that muscle strength fluctuated with extremes of temperature although myotonia continued to dominate his clinical picture and to be his main complaint.

At this stage, given the episodes of weakness, acetazolamide 125 mg twice daily was commenced in addition to an increased dose of mexiletine 200 mg three times daily. Despite up-titration, there was no therapeutic benefit from acetazolamide and it was discontinued.

Myotonic symptoms became progressively worse. At age 30, he required mexiletine 600–800 mg per day to manage the myotonia. He continued to describe substantial episodes of temporary weakness involving paralysis of the legs lasting 5–10 minutes and less well defined episodes of weakness affecting his hands.

Past medical history was otherwise notable for chronic lumbar back pain secondary to prior assault and a psychotic episode requiring long-term treatment with amisulpride at age 29.

The proband is the 2^nd^ oldest of 4 brothers from consanguineous parents (first cousins). He is related to his wife (further separated than first cousins) with whom he has one son who is clinically unaffected. There is limited pedigree information. It is unknown if the proband’s father or oldest brother are affected. He reports that his younger brother is symptomatic with difficulty standing after sitting for prolonged periods and repeated falls. His maternal uncle, paternal aunt and paternal uncle’s son report cramps and stiffness but they have not been reviewed at our centre.

Investigations demonstrated myotonia on electromyography (EMG) with a Fournier pattern type III on repetitive short exercise test (SET) (pattern seen in MC, SCM and normal controls). One of two long exercise tests (LET) produced a decrement in CMAP of 45% (positive test usually seen in periodic paralysis). Creatinine kinase was persistently mildly elevated between 771 and 966 IU/L. MRI imaging of the thighs and calves as well as neuroaxis at age 26 were normal.

**Figure 1:**
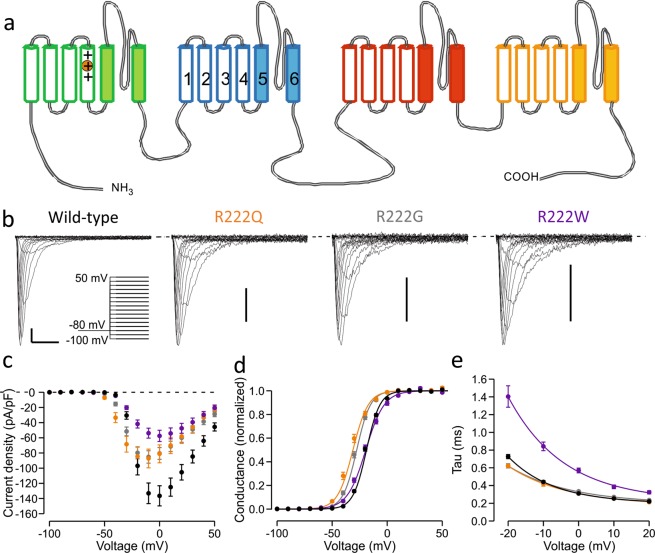
Location of the R222 residue and activation properties of R222 mutant channels. (**a**) Topological representation of Na_V_1.4 channel. The four repeats (I-IV) of the channel are shown in green, blue, red and yellow, respectively. Each repeat contains six transmembrane helices (1–6) of which the first four (open) form a voltage-sensing domain (VSD). Helices 5–6 (filled) of each repeat form the central ion-conducting pore. Fourth transmembrane helix (S4) contains several arginine residues. R222 is the second outermost arginine (R2) in the S4 helix of the VSD-I (circled). (**b**–**e**) Characterization of the activation properties of R222 mutant channels. Wild-type data is shown in black symbols, R222Q in orange, R222G in grey and R222W in purple. See Table 1 for numeric data. (**b**) Representative main pore currents in response to voltage steps between −100 mV to +50 mV in 10 mV increments. Voltage protocol is shown in insert. Scale bars: x-axis 2 ms, y-axis 25 pA/pF. (**c**) Mean current density. (**d**) Voltage dependence of activation. Solid lines show Boltzmann equation fits to mean Conductance-voltage data. (**e**) Time constant of inactivation following channel opening. Solid lines represent a fit an exponential curve fit to mean time constant-voltage data.

Next generation sequencing demonstrated a novel heterozygous variant in the *SCN4A* gene c.665G>A p.Arg222Gln (R222Q). The mutation affects a highly conserved arginine residue in the S4 helix of VSD-I (Fig. 1a) and is absent from the GNOMAD control database.

A heterozygous synonymous *CLCN1* variant was also found c.1650G>A p.(Thr550=). This *CLCN1* variant has been identified by our diagnostic service in other symptomatic patients with myotonia who are either homozygous for it or have a second pathogenic mutation in *CLCN1* (supplementary information in Horga *et*. *al* 2013^11^). Heterozygous carriers are reported to be unaffected. In addition to this pedigree, the c.1650G>A variant has subsequently been identified in other MC cases by our diagnostic service. In three patients it was found with a second *CLCN1* pathogenic mutation, and one patient is homozygous for c.1650G>A. Family members in the R222Q index case were not available to confirm reported clinical symptoms, nor was DNA available to interrogate segregation. The synonymous *CLCN1* variant creates a potential cryptic splice acceptor site and strengthens a previously existing GT cryptic splice donor present in the reference sequence (Supplementary Fig. 1).

Other mutations affecting the Na_V_1.4 residue R222 have been described in the literature in patients diagnosed with HypoPP. The R222G mutant channel conducts gating pore currents and reportedly caused HypoPP, although detailed clinical information was not available^9^. Mutation R222W has been found by us in two apparently unrelated HypoPP kindreds^8^. Brief clinical details were previously reported but detailed information is now available for one kindred and confirms a phenotype of HypoPP without any additional or unusual features (Supplementary Results, Supplementary Fig. 2).

### Characterization of the functional properties of wild-type and mutant Nav1.4 channels

To investigate the cause of phenotypic variability of the mutations affecting the same S4 arginine residue we compared the properties of the R222Q mutant showing a predominantly myotonic presentation with the HypoPP mutant channels R222W and R222G.

Patch clamp recordings did not reveal changes in current density for R222Q mutant channels compared to WT channels (Fig. 1, Table 1). For the HypoPP variant R222G current density was slightly but significantly reduced, while in cells expressing R222W it was less than half of that recorded in cells expressing wild-type channels. Voltage of half-maximal activation was significantly shifted to hyperpolarized voltages for R222Q and R222G channels, consistent with a gain of function, but was unaffected for R222W channels, though the slope of voltage dependence was less steep.

**Table 1:** Properties of main pore currents of wild-type and mutant channels.

Parameter	R222G	R222Q	R222W	WT
Activation	n = 27	n = 12	n = 16	n = 38
V_1/2_ (mV)	−28.4 ± 0.7***	−32.5 ± 1.1***	−19.9 ± 0.8	−19.5 ± 0.5
V_Slope_ (mV)	6.3 ± 0.2	7.5 ± 0.2**	8.7 ± 0.3***	6.3 ± 0.2
Peak current density	−86.3 ± 8.3*	−88.6 ± 12.7	−58.0 ± 7.4***	−138.7 ± 13.8
Fast Inactivation	n = 27	n = 12	n = 15	n = 38
V_1/2_ (mV)	−70.3 ± 0.7***	−71.8 ± 1.1***	−67.0 ± 1.3	−64.8 ± 0.4
V_Slope_ (mV)	4.8 ± 0.1	4.9 ± 0.2	4.4 ± 0.1***	5.2 ± 0.1
T_Recovery@−80mV_ (ms)	8.1 ± 0.4*** (n = 22)	9.2 ± 1.1* (n = 5)	6.4 ± 0.5 (n = 14)	5.9 ± 0.2 (n = 34)
T_Inactivation@0mV_ (ms)	0.33 ± 0.01	0.31 ± 0.01	0.57 ± 0.03***	0.31 ± 0.01
Slow Inactivation	n = 16	n = 6	n = 8	n = 26
V_1/2_ (mV)	−61.3 ± 1.5***	−66.5 ± 2.4***	−56.8 ± 1.6*	−51.0 ± 0.5
V_Slope_ (mV)	12.2 ± 0.4	9.9 ± 0.6	11.6 ± 0.6	11.3 ± 0.2

Data in the table was analysed using one-way ANOVA, except for peak current density and recovery time constant that were studied using Kruskal-Wallis ANOVA. Means were compared using Bonferroni post tests except for Kruskal-Wallis ANOVA test where clones were compared with Dunn’s multiple comparisons test. Statistically significant changes are indicated by asterisks (*p < 0.05, **p < 0.01, ***p < 0.001).

**Figure 2:**
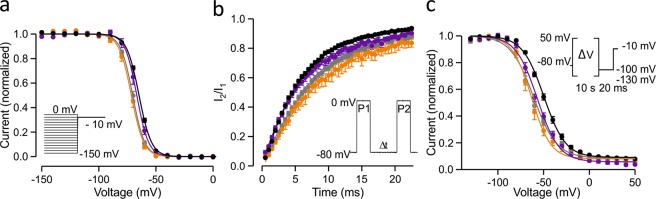
Inactivation properties of R222 mutant channels. Wild-type is shown in black symbols, R222Q in orange, R222G in grey and R222W in purple. See Table 1 for numeric data. Voltage protocols are shown in the insert and described in methods. (**a**) Voltage dependence of fast inactivation. (**b**) Recovery from fast inactivation at −80 mV. (**c**) Voltage dependence of slow inactivation. Solid lines show Boltzmann equation fits to mean current-voltage data (**a,c**) or exponential curve fits to mean recovery time course data (**b**).

The voltage dependence of fast inactivation was shifted in a hyperpolarizing direction for all R222 mutant channels but did not reach significance for R222W channels (Fig. 2, Table 1). Consistently, the rate of recovery from inactivation was slower for R222Q and R222G channels than for wild-type channels (Fig. 2, Table 1). The rates of onset of open-state inactivation were similar to wild-type at all voltages for R222G and R222Q channels, while the inactivation rate of R222Wchannels was slowed compared to wild-type (Fig. 1d, Table 1).

A significant hyperpolarizing shift in the voltage dependence of slow inactivation was measured for all mutant channels compared to wild-type (Fig. 2, Table 1).

### Characterization of gating pore currents of wild-type and mutant Nav1.4 channels

Studies to characterise the gating pore current in isolation were undertaken by blocking the main pore current using tetrodotoxin (TTX) in *Xenopus laevis* oocytes. The currents were studied with extracellular media containing sodium or a 1:1 mix of sodium and guanidinium (Gn^+^). A guanidinium moiety is present in the side chain of arginine. Guanidinium substitution has been shown to increase the amplitude of gating pore currents^12,13^ and can be used to analyse the presence of gating pore currents^14^.

The slope of the conductance of the linear leak current at depolarized voltages was the same for all the clones in Na^+^ solution suggesting this voltage range is likely to represent the closed state of the gating pore for all mutants. Accordingly, the slope of the conductance of the linear leak current was the same for all the clones studied in Na^+^/Gn^+^ solution.

**Figure 3:**
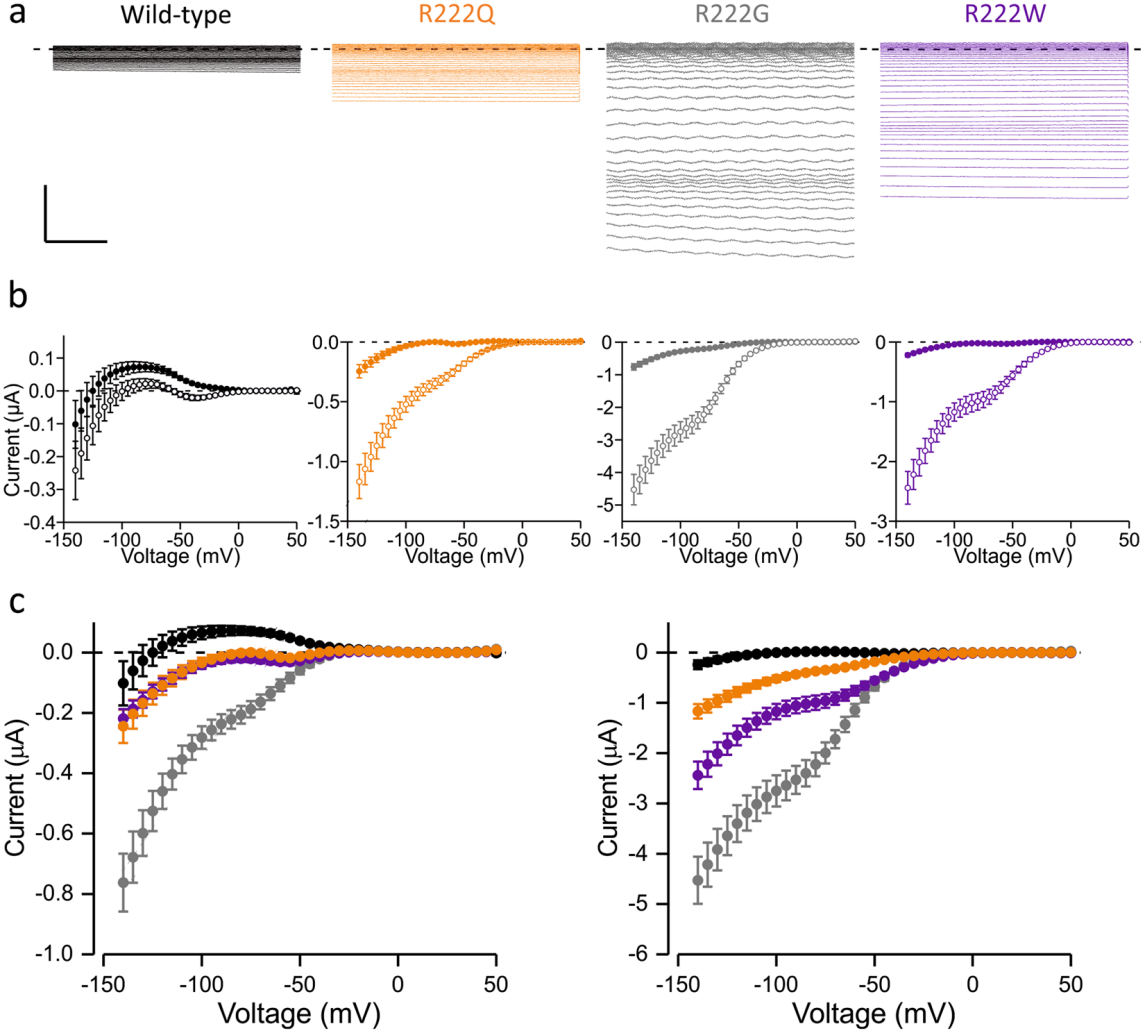
Gating pore currents. Wild-type data is shown in black, R222Q in orange, R222G in grey, R222W in purple. Data are mean ± SEM. (**a**) Raw leak currents in mixed sodium/guanidinium (Na^+^/Gn^+^)solution in response to voltage steps from −140 mV to +50 mV in 5 mV increments after blocking the central pore current with TTX. The last 200 ms of 300 ms pulse are shown. Scale bar: y axis: 1 µA, x axis: 50 ms. (**b**) I-V plots of leak-subtracted gating pore current traces in the presence of Na^+^ (solid symbols) or Na^+^/Gn^+^ (open symbols) solutions. Sample sizes: WT: n = 22, R222G: n = 25, R222Q: n = 24, R222W: n = 22. (**c**) Superimposed I-V plots for all the clones in Na^+^ (left) and Na^+^/Gn^+^ (right) solutions.

In the Na^+^ solution all R222 mutants expressed gating pore currents that differ from WT, with clear inward deflections in the hyperpolarized voltage range (Fig. 3). Leak-subtracted current amplitudes at −90 mV were 72 ± 17 nA, −237 ± 33 nA, −13 ± 13 nA, −25 ± 12 nA for WT, R222G, R222Q and R222W channels, respectively (p < 0.05 for R222Q, p < 0.01 for R222W and p < 0.001 for R222G, vs WT), indicating that the R222 mutants carry gating pore currents. To confirm that the difference in the current amplitude is due to gating pore currents we introduced guanidinium in the extracellular media. For all mutant channels, substitution of extracellular Na^+^ by Na^+^/Gn^+^ solution revealed a large inward current at negative voltages (p < 0.001). Introduction of guanidinium resulted in a statistically insignificant change in wild-type channel currents, with the current amplitude at −90 mV being 15 ± 20 nA. This clearly indicates that the R222 mutant channels carry hyperpolarization activated gating pore currents. The gating pore current amplitude at −90 mV was −2.5 ± 0.3 µA, −1.1 ± 0.2 µA and −0.4 ± 0.1 µA for R222G, R222W and R222Q channels, respectively. The amplitude was smaller in R222Q channel found in the patient with myotonia than in R222W (P < 0.05) and R222G channels (p < 0.001) associated with HypoPP. The difference in gating pore current amplitude is unlikely to be due to different expression levels of mutant channels as the mean peak main pore current before TTX block was the same for all variants.

### Neurophysiological investigations support pathogenicity of synonymous *CLCN1* variant

A synonymous *CLCN1* variant c.1650G>A p.(Thr550 = ) was also found in the proband. Genetic data from other pedigrees suggests that this variant is associated with recessive myotonia congenita^11^. We sought further evidence for the pathogenicity of the variant using muscle velocity recovery cycle (MVRC) analysis, comparing the data for the proband against our previously published data in patients with SCM (n = 9) and PMC (n = 8) (*SCN4A* mutations)^15^, and Myotonia Congenita (n = 11, *CLCN1* mutations: autosomal dominant n = 5, recessive n = 6, selecting only those who were not on sodium channel inhibitors) and normal controls (NC, n = 26, who were age and sex matched for the myotonia groups)^16^.

**Figure 4:**
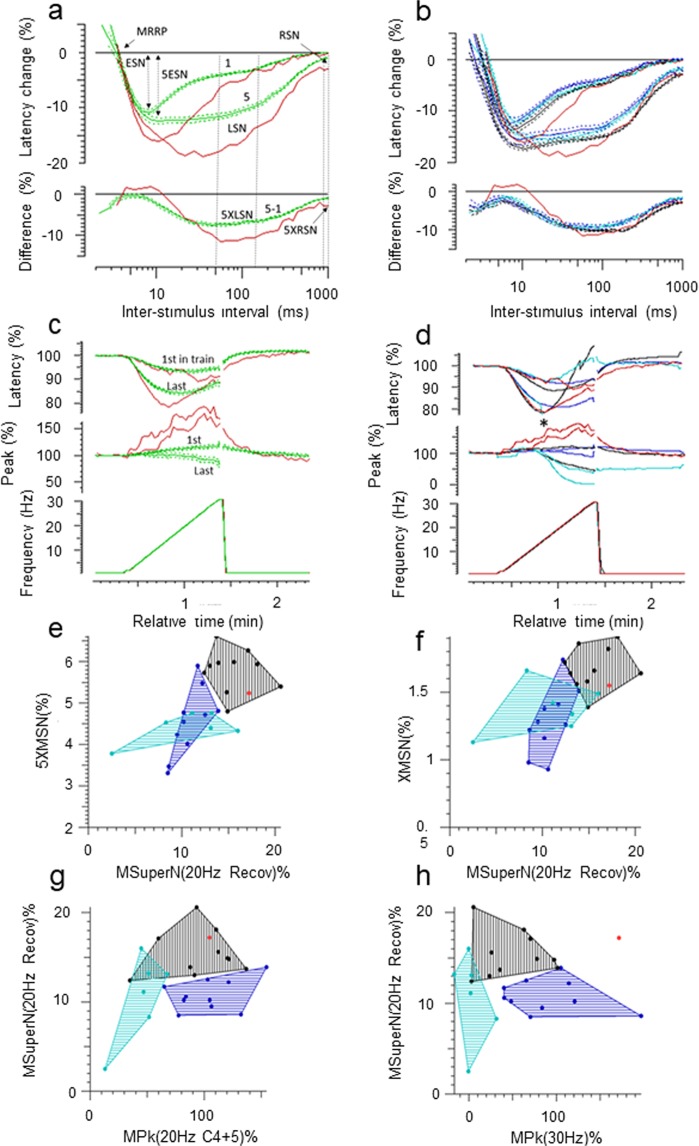
(**a**) Muscle velocity recovery cycles (MVRC) to one and five conditioning stimuli. Normal controls (green, mean ± SE, n = 26), R222Q (red). The muscle relative refractory period (MRRP), early supernormality (ESN), and late supernormality (LSN), 5ESN, residual supernormality (RSN) and extra residual supernormality after 5 conditioning stimuli (5XRSN) are as defined in the text. (**b**) Superimposed MVRCs of Sodium channel myotonia (blue, n = 9), Paramyotonia congenita (cyan, n = 8), Myotonia congenita (black, n = 11), and R222Q patient (red). Data is mean ± SE. (**c**) Ramp. Normal controls (green, n = 26, mean ± SE), R222Q (red). d. Ramp. Groups superimposed. For clarity, only means are shown. Sodium channel myotonia (blue, n = 9, mean), Paramyotonia congenita (cyan, n = 8, mean, * marks where the latency could no longer be measured because of the loss of amplitude), Myotonia congenita (black, n = 11, mean), R222Q patient (red). (**e,f**) Plots of excitability measurements taken from the MVRC, ramp, and repetitive stimulation data, showing R222Q (red) as an outlier to the SCM (blue) and PMC (cyan) groups, and either lying with the MC (black) group (**e**–**g**) or as an outlier for all 3 groups. (**h**) 5XMSN: extra mean supernormality with 5 conditioning stimuli; XMSN: extra mean supernormality after 2 conditioning stimuli; MSuperN(20 Hz Recov): early supernormality following 6 minutes of intermittent 20 Hz stimulation during the repetitive stimulation protocol; MPkf(20 Hz C4 + 5): amplitude of the first in 20 Hz train for MVRC cycles 4 and 5 during the repetitive stimulation; MPk 30 Hz: amplitude for the last in the 30 Hz train during the ramp (for more details, please see Tan *et al*., 2014, and 2018).

The results are shown in Fig. 4. Compared with healthy controls, the patient’s recording shows a) an increased early supernormality following one and 5 conditioning stimuli (ESN, 5ESN, which are the maximum percentage decrease in latency for interstimulus intervals <15 ms), and which reflects the depolarising afterpotential, b) an increased late supernormality (LSN, the mean percentage decrease in latency for interstimulus intervals between 50–150 ms) for one and 5 conditioning stimuli (5XLSN) which reflects the depolarising effect of potassium accumulation in the t-tubules, and c) an increased residual supernormality (mean percentage latency decrease for interstimulus intervals 900–1000 ms) following 5 conditioning stimuli (5XRSM, Fig. 4a) representing the residual depolarisation of the membrane at the end of the sweep. The muscle relative refractory period (MRRP) was no different from controls, and was normal.

When compared to the means of the patients with SCM, PMC and MC (Fig. 4b), the patient’s LSN exceeded the means ± SEs for all three groups. He had an increased 5XRSM which was of similar magnitude to that seen in SCM, PMC and MC. In the Ramp, the reduction in latency for the last in train was greater than in NC (Fig. 4c, top trace), but mirrored the findings in the MC group at lower ramp frequencies (Fig. 4d, top trace). At higher ramp frequencies, the latency of the last in train diverged from the MC group, eventually overlapping with NC. Immediately after cessation of the ramp the latency remained reduced compared with NC as well as the other myotonia groups. The R222Q patient ramp measurements were quite different from those of the PMC patients in whom the latencies for the last in train could not be tracked (*) at frequencies higher than 15–20 Hz because of a rapid loss in the amplitude of the response, due to depolarisation of the membrane to the point of inexcitability with rapid trains of action potentials (Fig. 4d)^15^.

The amplitude (Peak) of the patient’s responses (4c and d, middle trace) showed a progressive increase during the ramp which was not seen in NC or other patient groups. The progressive increment in amplitude with increasing frequency in the proband contrasts markedly with the dramatic fall in amplitude in the PMC group.

Scatter plots of the patient’s muscle excitability measurements showed him to be an outlier compared with SCM and PMC patients on numerous excitability measures derived from the MVRC, Ramp and Repetitive stimulation protocols, some of which are illustrated in (Fig. 4e–h) (for more details of these measurements, please see Tan *et al*. 2014 and Tan *et al*. 2018). In some of these measurements, the patient’s data more closely resembled the MC group (Fig. 4e–g), in others, his results were an outlier for all three myotonia groups (Fig. 4h).

## Discussion

We report a patient with a predominantly myotonic phenotype associated with mutation R222Q in the skeletal muscle sodium channel Na_V_1.4. In contrast, other substitutions of the same S4 arginine residue have been found in patients with HypoPP^8,9^. To assess the mechanism of this phenotypic variability we performed a functional analysis that revealed enhanced activation for R222Q channels, consistent with myotonia, but also loss of function features such as enhanced fast and slow inactivation. Additionally, R222Q channels conduct gating pore currents, a defect associated with HypoPP. The differences in clinical presentation, however, could not be assigned only to differences in biophysical characteristics of the three R222 variants as the R222Q proband carried a concomitant synonymous *CLCN1* variant. MVRC analysis suggests that this *CLCN1* variant is not silent and contributes to the severe myotonia in the patient.

The clinical classification of the patient’s phenotype prior to genetic investigations was challenging. He clearly presented with a myotonic disorder and this was initially classified as MC. The age of onset, predominant complaint of leg symptoms, recurrent falls and normal repetitive SET were all in keeping with this. The episodes of leg weakness however, seemed more defined and of longer duration than the transient weakness which usually improves with repetition that can sometimes be demonstrated in MC and overall we felt these were more in keeping with the weakness described by patients with periodic paralysis. His positive LET would also be consistent with this.

The functional features of the pore domain of the R222Q channel, associated with predominant myotonia, and of the R222G channel, associated with HypoPP, were very similar. Both channels show left-shifted voltage dependence of activation, and fast- and slow inactivation, although the amplitude of the shifts was slightly larger for the R222Q variant. Current density was similarly reduced for both channels, but reached significance only for R222G channels, probably due to larger sample size. It is unlikely that the distinct clinical presentations arise from these minor quantitative differences in channel dysfunction. Instead, the difference may arise from the amplitude of the gating pore currents at resting membrane potential, which was 5-fold larger for the HypoPP mutant R222G than for the R222Q mutant in Na^+^/Gn^+^ solution. As reported for other arginine neutralizing mutations in Ca_V_1.1 and Na_V_1.4, the large amplitude of the gating pore current of R222G channels is likely to underlie the reported HypoPP phenotype of that patient^4,9,10^. It was not reported if the R222G patient had clinical or EMG myotonia.

In contrast to R222Q channels, the R222W mutant showed significantly reduced main pore currents. In addition, the rate of open state inactivation was slowed, a gain of function feature associated with myotonia but not with HypoPP. R222W channels also displayed prominent gating pore currents. The gating pore currents depolarize the muscle, particularly during hypokalaemia that reduces the hyperpolarizing potassium currents through Kir2.1 channels at resting membrane voltages^14,17^. Although the R222Q gating pore current amplitude is smaller than in HypoPP mutant channels, it is expected that the gating pore current of R222Q would increase the probability of finding the muscle in a stabilized depolarized state, and consequently contribute to the occurrence of lower limb episodic muscle weakness in the patient. However, the MVRC findings of a normal muscle relative refractory period and large early supernormality in our patient suggests that the resting membrane potential was not depolarised at least at the time this patient was studied.

In addition to Na_V_1.4 R222Q mutation, the proband carries a synonymous *CLCN1* c.1650G>A variant. Clinical, genetic and muscle excitability recordings suggest that the synonymous *CLCN1* c.1650G>A variant is not functionally silent in our patient, and may act as a modifier to exacerbate the severity of myotonia. Other heterozygous *CLCN1* mutations are known to modify the phenotype of sodium channel myotonia^18–20^. The *CLCN1*c.1650G>A variant was previously reported in a pedigree with homozygous or compound heterozygous patients^13^. It was since also found in four additional compound heterozygous or homozygous cases with MC. These data strongly support association of *CLCN1*c.1650G>A with recessive MC. Analysis of effects of c.1650G>A variant in splicing using Alamut suggests that the variant creates a potential cryptic splice acceptor site and strengthens a previously existing GT cryptic splice donor present in the reference sequence (Supplementary Fig. 1). Potential effects of the synonymous variant on splicing need to be experimentally confirmed from muscle biopsy or by using a mini-gene assay.

In addition, many muscle excitability parameters of the proband were outliers for both the SCM and PMC groups and more closely aligned to changes seen in MC (Fig. 4). The degree of increase in the ESN and LSN, which was large even when compared with the SCM, PMC and MC groups is not easily accounted for solely by the Nav1.4 channel dysfunction in our patient, but may possibly be explained by the combined effects of a reduced resting chloride conductance, and an increased sodium current. These data strongly support the pathogenicity of *CLCN1* c.1650G>A variant. The increased residual supernormality seen in the proband following five conditioning stimuli was of an order similar to that seen in SCM, PMC and MC, and would explain the propensity for myotonic discharges. The progressive increase in amplitude during the ramp was unusual, and could be explained by recruitment of muscle fibres adjacent to those being directly stimulated, due to the hyperpolarised shift in Na_V_1.4 channel activation caused by the R222Q mutation. At the end of the ramp, the latency remained faster than controls and the other myotonia patient groups, suggesting a greater propensity for spontaneous discharges after activation.

Pharmacological block of the Na-K-2Cl transporter by bumetanide has been shown to have beneficial effects on mouse models of HypoPP^21,22^, probably by hyperpolarizing the equilibrium potential of chloride. In addition, the ClC-1 channel blocker 9-AC is able to hyperpolarize a depolarized muscle and can prevent depolarization if given prior to hypokalaemia^23^, suggesting that pharmacological manipulation of the chloride conductance can be used to prevent muscle depolarization and ensuing paralysis. Consequently, it is possible that the presence of the MC-associated *CLCN1* mutation in the proband modulates or reduces the duration or severity of episodes of paralysis.

It is also of note that many HyperPP mutations show attenuated slow inactivation^24^. This has been suggested to be essential to allow sodium influx during prolonged depolarization. The R222Q mutant channels display enhanced slow inactivation, which may instead lead to a reduction in sodium current during prolonged depolarization and contribute to the absence of hyperkalaemia-induced episodes of paralysis in the patient.

The voltage dependence of fast inactivation has been shown to shift for R222W channels in the *Xenopus* oocyte system^25^ while in our experiments in HEK293 cells the shift in the voltage dependence of inactivation did not reach statistical significance. However, in the *Xenopus* oocyte system, we see a larger amplitude shift in the voltage dependence of fast inactivation for all three R222 mutant channels (not shown). Sodium channel β-subunits modulate the rate and the voltage dependence of fast^26,27^ and slow^28^ inactivation. Discrepant fast inactivation defects in HEK293 cells and *Xenopus* oocyte systems may rise from different expression of β-subunits in these expression systems or from the absence of co-transfected β-subunit in our HEK293 cell experiments.

Our analysis of the Na_V_1.4 R222 variants shows that biochemical properties of the residue substituting this second most extracellular arginine (R2) of S4 helix substantially affect the biophysical features of the mutant channel. Introduction of glycine or glutamate residue apparently destabilizes the resting state of the channel (left shift in the voltage dependence of activation), probably by destabilizing the resting state of the VSD-I. R2 mutant Kv channels display similar shifts in channel activation^29,30^. Resting state structure of Na_V_AB channel^31^ and molecular modelling of K_V_10.2 channel^29^ suggest that R2 arginine stabilizes the resting state of the voltage sensor with ionic interactions between the arginine and negatively charged residues within the helices S2 and S3 (Supplementary Fig. 3). Subsequently a mutation of this arginine may promote channel activation by disrupting these interactions^29,31^. Our data shows that activation of R222W was less disrupted than for glycine and glutamate substituted channels suggesting that the tryptophan residue may form interactions that stabilize the resting state of VSD-I. However, these interactions differ from those in wild-type channel as R222W channel is unable to prevent gating pore current leak currents. Consistent with modelling data for Na_V_AB R2G mutant channel^32^, R222G, but also R222W and R222Q channels, can provide an aqueous path through the membrane that allows depolarizing gating pore leak currents.

In summary, we have shown that the biophysical features of different mutations affecting the second arginine of the Na_V_1.4 VSD-I S4 helix cannot fully explain why the carrier of a R222Q mutation presents with predominant myotonia while carriers of R222W and R222G present with HypoPP. Muscle excitability recordings suggest that a concomitant synonymous *CLCN1* variant in R222Q patient is not silent, but rather exacerbates myotonia. We speculate it may also protect the muscle against fulminant paralytic episodes.

Mexiletine is the first choice drug for non-dystrophic myotonia for both MC and sodium channel myotonia^33^. The patient with concomitant Na_V_1.4 R222Q and *CLCN1* c.1650G>A mutations benefits from mexiletine therapy, consistent with predominant myotonic presentation. Mexiletine was found effective also for other cases with concomitant gain of function Na_V_1.4 mutations and loss of function ClC-1 mutations^18^. Thus, it should be considered as first line therapy for patients with concomitant *SCN4A* and *CLCN1* mutations associated with myotonia.

## Methods

### Ethical approval

Ethical approval to use clinical data has been attained from the Joint National Hospital for Neurology and Institute of Neurology Research Ethics Committee as part of the Investigation of human neurological ion channel and episodic neurological disorders study. For neurophysiological research an informed written consent was obtained from all patients and controls according to the Declaration of Helsinki. The study was approved by the London - Westminster Research Ethics Committee. Oocytes were isolated from *Xenopus laevis* in accordance with the UK Animal (Scientific Procedures) Act 1986. The procedures are approved by the UCL Biological Services and the UK Home Office.

### Patient data

All patient data were obtained as part of routine clinical care at the National highly specialised service for skeletal muscle channelopathies at the National Hospital for Neurology and Neurosurgery (NHNN). Neurological examination, neurophysiology studies (NCS/EMG/LET protocol) and genetic analysis were performed as part of the routine diagnostic process. The patient subsequently consented to additional neurophysiology tests (muscle excitability) performed as part of a research project.

### Genetic analysis

DNA was isolated from peripheral blood using standard methods. *SCN4A* coding regions and exon-intron boundaries were amplified by polymerase chain reaction (PCR). Genetic analysis of ion channel genes *SCN4A*, *CACNA1S*, *KCNJ2* and *CLCN1* was performed at the Neurogenetics Unit, NHNN, as provided by the Channelopathy Highly Specialized National Service for rare diseases. Samples underwent Next-Generation Sequencing on an Illumina HiSeq following enrichment with an Illumina custom Nextera Rapid Capture panel (Illumina, Inc., San Diego, CA).

### Molecular biology and cell preparations

Site directed mutagenesis of human and rat *SCN4A* constructs was performed using the QuickChange kit (Agilent Technologies). Successful mutagenesis was confirmed by Sanger sequencing. *In vitro* transcription of the rat clone was performed with the mMessage mMachine kit (Ambion). HEK293 cells were cotransfected with h*SCN4A* (500 ng) and GFP (50 ng) cDNAs using 1.5 µl Lipofectamine 2000 (Gibco) on a 1.9 cm2 dish.

*Xenopus laevis* oocytes were isolated, stored and injected as described in Luo, *et al*.^34^ and in the supplementary file.

### Molecular electrophysiology

The main pore currents were studied with whole cell patch clamp recordings from HEK293 cells 48 hours following transfection. Data were filtered at 5 kHz and sampled at 50 kHz with Axopatch 200B, Digidata 1440B and pCLAMP™ software (Molecular Devices). Bath solution was (in mM): NaCl 145, KCl 4, CaCl_2_ 1.8, MgCl_2_ 1, and HEPES 10 (pH 7.35). Electrodes were filled with intracellular solution (in mM): CsCl 145, NaCl 5, EGTA 10, HEPES 10 (pH 7.4) and had a resistance of 1–3 MΩ. The calculated liquid junction potential was −4.4 mV but was not corrected for. Series resistance was compensated ≥70% to keep the voltage error below 5 mV. Holding potential was −80 mV, leak and capacitance subtraction was performed online using a -*P*/4 procedure for all protocols except for slow inactivation.

Patch clamp voltage protocols are described in Luo, *et al*.^34^ and in the supplementary file and illustrated in figures.

The voltage of half-maximal activation or inactivation (V_1/2_) and the slope factor (V_Slope_), were measured by fitting with a Boltzmann equation (Y = A + (B-A)/(1 + exp((V-V_1/2_)/V_Slope_)) where Y is the conductance or the current, and A and B are the minimum and maximum amplitudes of the fit.

Gating pore currents were studied in *Xenopus laevis* oocytes using two-electrode voltage clamp using GeneClamp 500B amplifier, Digidata 1200 digitizer and pCLAMP™ software (Molecular Devices). Electrodes had a resistance of ≥ 0.1 MΩ when filled with 3 M KCl. Bath Na^+^ solution contained (in mM): 120 NaMeSO_4_, 1.8 CaSO_4_, 10 HEPES, pH 7.4. Oocytes were clamped to voltages ranging from −140 to + 50 mV in 5 mV increments from a holding potential of −100 mV, filtered at 1 kHz and sampled at 10 kHz. TTX 1–2 μM was added to block Na^+^ currents through the main channel pore and replacing 50% of NaMeSO_4_ with guanidine sulfate (Na^+^/Gn^+^ solution) was used to study the presence and properties of gating pore currents. Gating pore currents were measured as the average steady state current in the last 100 ms of the test pulse. Linear leak currents were estimated by fitting a straight line to the current-voltage data in the range +10 mV to +30 mV. The extrapolated leak current was subtracted from the raw current data.

### Muscle velocity recovery cycle analysis

Changes in membrane potential that occur during and after a muscle action potential are reflected by changes in muscle membrane excitability. This can be measured using a protocol first described by Z’Graggen and Bostock^35^ and further by Tan, *et al*.^16^, which allows *in vivo* assessment of muscle ion channel function by monitoring changes in the amplitude and latency of responses of muscle fibres to a test stimulus given at set times following conditioning stimuli.

Recordings were performed in the distal portion of tibialis anterior using a monopolar stimulating needle and a concentric needle for recording. Stimulation and recording were controlled by Qtrac software (Digitimer Ltd, Welwyn Garden City, United Kingdom).

Three main stimulation paradigms are used: a) *muscle velocity recovery cycle (MVRCs)*, where responses are recorded following 1, 2, and 5 conditioning stimuli (10 ms apart). The interstimulus interval between the last conditioning stimulus and the test stimulus is reduced from 1,000 ms to 1.4 ms, (b) a *ramp* of progressively longer trains of stimuli from 1–30 Hz, to characterise the effects of progressive muscle activation, and c) *repetitive stimulation*, where the effects of intermittent high frequency trains of stimulation are studied using repeated 1-second trains of stimuli at 20 Hz for 6 minutes. Repeated short MVRCs are recorded before, during and after the repetitive stimulation, as previously described^16,36^.

### Data analysis

Effects of mutation on splicing were analysed using Alamut Visual v2.7.1 (Interactive Biosoftware, Rouen, France). Current recordings were analysed using Clampfit 10.7, OriginPro 2016 and GraphPad softwares. All data is presented as mean ± SEM. Patch clamp data was analysed using one-way ANOVA, except for open-state inactivation data that was studied using two-way ANOVA, and the peak current density and recovery time-constant data that were analysed using Kruskal-Wallis ANOVA. The gating pore current amplitude data were compared at −90 mV using Kruskal-Wallis ANOVA. Means were compared using Bonferroni post tests for one and two way ANOVA analyses or using Dunn’s multiple comparison tests for Kruskal-Wallis analyses. Significance was established at p < 0.05. Some of the raw data for R222W and wild-type main pore currents and for R222W, R222G and wild-type gating pore currents has been published^14^.

## Supplementary information

Supplementary Info

## Data Availability

The datasets generated during and/or analysed during the current study are available from the corresponding author on reasonable request.
