# Supplementary material for: Myotonia in a patient with a mutation in an S4 arginine residue associated with hypokalaemic periodic paralysis and a concomitant synonymous CLCN1 mutation
Source: Sci Rep. 2019 Nov 26;9:17560. doi: 10.1038/s41598-019-54041-0 (PMC6879752; doi:10.1038/s41598-019-54041-0)
Supplement: Supplementary file 1 — Supplementary Info [file 41598_2019_54041_MOESM1_ESM.pdf]

Myotonia in a patient with a mutation in an S4 arginine residue associated with hypokalaemic periodic paralysis and a concomitant synonymous *CLCN1* mutation

Michael G Thor[a], Vinojini Vivekanandam[a], Marisol Sampedro Castañeda[a,£], S Veronica Tan[a,c], Karen Suetterlin[a], Richa Sud[a], Siobhan Durran[a], Stephanie Schorge[b,$], Dimitri M Kullmann[b], Michael G Hanna[a], Emma Matthews[a], Roope Männikkö[a,*]

Supplementary information

Supplementary Results

Patient data for HypoPP patient carrying the Nav1.4 R222W mutation

The proband had an unremarkable early history including normal motor milestones. First symptoms occurred at age 15 when after 24 hours of sitting on a coach, he found that his thighs felt heavy and walking unaided was difficult. Limb weakness progressed to quadriparesis, with speech and swallowing preserved. At presentation to the hospital, his serum potassium level was recorded at 1.3mmol/L and corrected. Over the subsequent 24 hours, strength gradually improved, first in the arms and then the legs. After 48 hours, he was able to walk and on day eight had returned entirely to normal strength and function.

He had a similar attack two weeks later, waking during the night unable to move his arms and legs. On this occasion, he took oral potassium prescribed after the first event and was able to walk by lunchtime. Subsequent neurophysiology investigations with long exercise tests demonstrated a decrement in CMAP of 46%, consistent with a diagnosis of HypoPP. He felt his attacks were manageable with intermittent potassium supplementation and avoidance of triggers, including excessive exercise.

The family pedigree is presented in supplementary figure 1. His sister developed symptoms at age 14 with an episode of weakness manifesting as difficulty walking after playing football. Symptoms settled within 24 hours. Exertion remains her predominant trigger and she manages this with potassium supplementation after playing sport. The proband's father is also affected, with very occasional attacks of weakness, triggered predominantly by carbohydrate rich meals. A paternal uncle is also affected, requiring daily potassium supplementation. The proband's mother is unaffected.

Next Generation Sequencing identified an *SCN4A* mutation c.664C>T; p.Arg222Trp. This mutation has been described in several unrelated families with Hypokalaemic periodic paralysis[1].

Supplementary methods

Molecular biology and cell preparations

Xenopus laevis ovarian lobes were obtained according to procedures approved by the UCL Biological Services and the UK Home Office. Oocyte follicular layer was enzymatically digested using Collagenase A (1-2 mg/ml, Roche) in oocyte Ringer (in mM: NaCl 82.5, KCl 2, $MgCl_2$, HEPES 5, pH 7.5-6) and stored in modified Barth's Solution (in mM: NaCl 87.1, KCl 1, $MgSO_4$ 1.68, HEPES 10, $NaNO_3$ 0.94, $NaHCO_3$ 2.4, $CaCl_2$ 0.88, pH 7.4) supplemented with penicillin (50 U/ml), streptomycin (50 μg/ml) and amikacin (100 μg/ml) at 14-18 °C. Healthy stage 5-6 oocytes were injected with mRNA for rat *SCN4A* and rat *SCN1B* in 1:1 weight ratio (~50 ng each).

Molecular electrophysiology

Peak currents in response to test voltages and in response to the tail voltage were used to measure the voltage dependence of activation and inactivation, respectively. Peak current in

response to test voltages was divided by test voltage subtracted by reversal voltage to derive peak conductance. Reversal voltage was estimated for each cell by fitting a straight line to current-voltage data in the range +20 mV to +50 mV and extrapolated to the voltage where current is zero. The mean E_{Rev} for wild-type cells was 73 ± 2 mV and, unsurprisingly, did not statistically differ for any of the VSD mutant channels. To estimate the time course of open state fast inactivation a double exponential function was fitted to the current decay following activation. The main component of the double exponential function was used for analysis.

The time course of recovery from fast inactivation was measured by applying an inactivating pulse for 20 ms at 0 mV, recovery-pulses of increasing duration to -80 mV, followed by second test pulse to 0 mV. The ratio of peak currents in response to second versus the first test pulse was plotted against the duration of pre- or recovery-pulse and fitted with a single exponential equation.

To measure slow inactivation the stimulus consisted of 10 s pre-pulse steps to voltages ranging from -130 mV to +50 mV in 10 mV increments, a 20 ms pulse to -100 mV to recover channels from fast inactivation and a test pulse to -10 mV. Peak currents in response to the test pulse were measured and plotted against pre-pulse voltage. It is of note that the recovery from inactivation was slower for R222Q and R222G channels compared to wild-type channels measured at -80 mV. We did not study the recovery rate at -100 mV and cannot say if the mutant channels recover from inactivation to the same extent as the WT channels during the 20 ms recovery step to -100 mV. However, potential small differences in the extent of recovery from fast inactivation should affect only the estimation of the peak amplitude but not of the voltage dependence of slow inactivation.

Supplementary References

1. Matthews, E. *et al.* Voltage sensor charge loss accounts for most cases of hypokalemic periodic paralysis. *Neurology* **72**, 1544-1547 (2009).
2. Wisedchaisri, G. *et al*. Resting-State Structure and Gating Mechanism of a Voltage-Gated Sodium Channel. *Cell* **178**, 993-1003 (2019).

Supplementary Figures

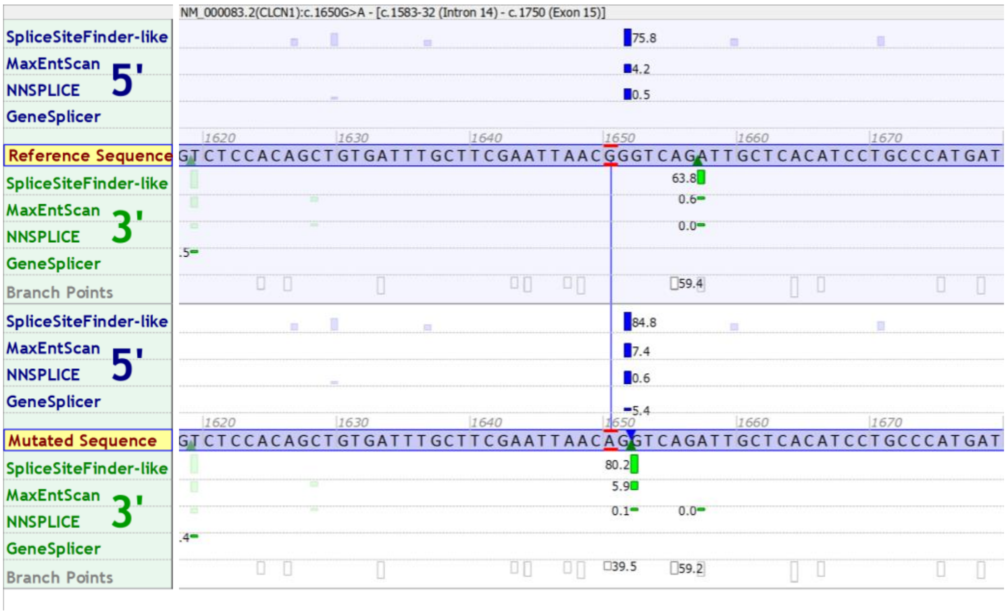

Supplementary figure 1. Output from the splicing analysis function in Alamut for CLCN1 wild-type (top) and c.1650G>A (bottom) variant. Blue bars indicate potential donor splice sites, green bars acceptor splice sites and white bars branch points. The variant creates a potential cryptic splice acceptor site and strengthens a previously existing cryptic splice donor present in the reference sequence.

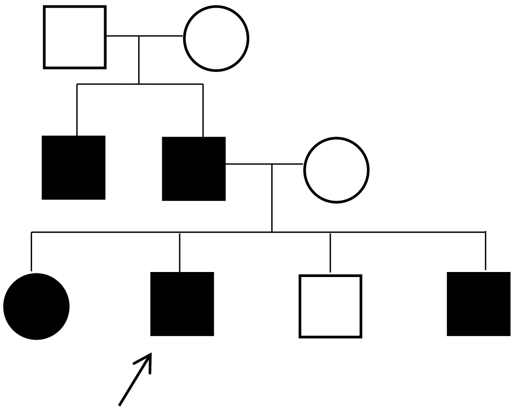

Supplementary figure 2. The family pedigree of HypoPP patient carrying R222W mutation. The proband is indicated with an arrow.

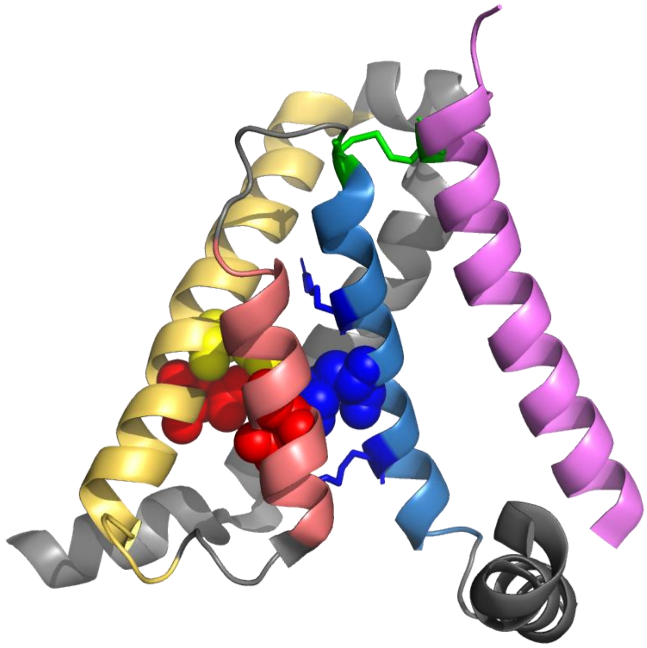

Supplementary figure 3. Structural features of R2 residue in Na_VAB cryo-EM structure. R2 residue (R102) in Na_VAB structure[2] (blue sphere) is shown in blue S4 helix. R1 (R99) and R3 (R105) are shown in sticks. Negative counter charges in S2 (yellow) and S3 (salmon) helices are shown in red spheres. R2 makes a charge-pair interaction with E59 on S2. Phe56 in hydrophobic constriction site is shown in yellow sphere. Green sticks show disulphide bond between G94C in S4 and Q150C in S5 helix (pink) of neighbouring subunit.
